# Reactive oxygen species in spermatozoa: methods for monitoring and significance for the origins of genetic disease and infertility

**DOI:** 10.1186/1477-7827-3-67

**Published:** 2005-11-29

**Authors:** Mark A Baker, R John Aitken

**Affiliations:** 1 The ARC Centre of Excellence in Biotechnology and Development, Reproductive Science Group, School of Environmental and Life Sciences, University of Newcastle, Callaghan, NSW 2308, Australia

## Abstract

Human spermatozoa generate low levels of reactive oxygen species in order to stimulate key events, such as tyrosine phosphorylation, associated with sperm capacitation. However, if the generation of these potentially pernicious oxygen metabolites becomes elevated for any reason, spermatozoa possess a limited capacity to protect themselves from oxidative stress. As a consequence, exposure of human spermatozoa to intrinsically- or extrinsically- generated reactive oxygen intermediates can result in a state of oxidative stress characterized by peroxidative damage to the sperm plasma membrane and DNA damage to the mitochondrial and nuclear genomes. Oxidative stress in the male germ line is associated with poor fertilization rates, impaired embryonic development, high levels of abortion and increased morbidity in the offspring, including childhood cancer. In this review, we consider the possible origins of oxidative damage to human spermatozoa and reflect on the important contribution such stress might make to the origins of genetic disease in our species.

## 1. Introduction – origins of genetic disease

The maintenance of genetic integrity in the male germ line has major repercussions for conception, the progress of pregnancy and, ultimately, the health and well-being of the progeny [1]. The human male contributes heavily to germ line mutations [2], and as such, is responsible for most of the dominant genetic diseases observed in our species. Indeed, in some cases, such as multiple endocrine neoplasia or achondroplasia (short-limbed dwarfism), the phenotype is invariably the result of mutations that can be traced back to the paternal germ line [2]. Epidemiological data also suggest that paternally derived genetic damage may contribute significantly to the aetiology of cancer in children and young adults [1,2].

These observations raise important questions about the aetiology of genetic damage in the male germ line and the causal links that exist between the induction of such damage and the inheritance of many childhood diseases. As early as 1912, Wilhelm Weinberg (cited in [2]) reported that children with dominant achondroplasia born to normal parents were among the last-born children in the family. Later work by Penrose [3] suggested that the effect observed by Weinberg was not actually correlated with birth order, nor surprisingly, maternal age. Rather, achondroplasia was a disease associated with paternal age. The implications of these findings were vast. Why is it that a much greater mutation rate apparently exists in the male germ line compared to the female? And why are several X-linked recessive and autosomal-dominant diseases correlated with paternal age?

The current consensus is that replication errors are the probable cause of such mutations as a consequence of the higher number of cell divisions involved in generating a spermatozoon (approximately 840 for a 50 year old male) as opposed to an ovulated egg (approximately 22 divisions regardless of age). If this is the case, then the mutations would have to be generated and retained in mitotically active spermatogonia. In the case of Apert syndrome, for example, there is good reason to believe that the causative mutation (in this case, predominantly a 755C-G transversion in the FGFR-2 gene) arises in spermatogonia and is selectively retained in the germinal epithelium because the mutant germ cells enjoy an unspecified selective advantage [4].

However, the replication-error hypothesis does not hold for all dominant genetic mutations; achondroplasia being a particular case-in-point. By taking sperm DNA from donors of different ages, Tiemann-Boege and colleagues [5] have examined the frequency of nucleotide substitutions in the fibroblast growth factor receptor 3 (FGFR3) gene that are the predominant cause this condition. They concluded that the magnitude of the increased mutation frequency associated with paternal age was insufficient to explain the exponential rise in the incidence of achondroplasia in the offspring [4]. Of the several possible hypotheses that have been advanced to explain this situation, one of the most plausible proposes that replication error is not responsible for the mutations causing for this disease. Rather, age-related premutational lesions may have occurred in these cells that are converted to the authentic mutation (most commonly a glycine to arginine substitution at codon 1,138) following fertilization, as a consequence of aberrant DNA repair in the zygote [5,6].

In order for the premutational lesion hypothesis to account for a mutation that is present in every cell of the body, rather than a mosaic, this putative aberrant repair would have to precede the S phase of the first mitotic division. The oocyte is well endowed with poorly characterized enzymes for effecting DNA repair [7,8], including enzymes that are known to be active prior to the initiation of DNA synthesis during S-phase, at a time when the sperm chromatin is undergoing decondensation [9]. If an oocyte is fertilized with DNA-damaged spermatozoa, these G1-associated DNA repair mechanisms become activated, leading to a dramatic suppression of pronuclear DNA-synthesis via a p53 -dependent mechanism [9].

Of course, aberrant repair of DNA damage in the oocyte could account for a wide variety of genetic aberrations in embryos generated from DNA damaged spermatozoa, not just point mutations. Thus, exposure of spermatozoa to xenobiotics or X-irradiation is known to induce dominant lethal effects (post implantation pregnancy loss) and heritable translocations in the embryos of mated females as a consequence of chromosome mutations (breaks and rearrangements) as well as specific locus mutations [10,11] that could be the result of aberrant repair in the oocyte. If this mechanism is of fundamental importance in the causation of genetic disease, it places emphasis on discovering both the nature and extent of DNA damage in spermatozoa and the fidelity of the repair processes activated in the oocyte. Interestingly, there appear to be profound genetic differences in the capacity of oocytes to repair DNA damage introduced by the fertilizing spermatozoon [12]. However, what could be causing the DNA damage in spermatozoa? A possible clue might be found in the type of mutation most commonly observed in cases of achondroplasia.

As indicated above, the mutation seen in a vast majority of achondroplasia patients is a CG-AT transition in the FGFR3 gene. Since this is the most common base substitution observed following oxidative damage to DNA [13,14], it is plausible that the lesions responsible for the initiation of aberrant DNA repair in the oocyte are oxidative in nature. Such a hypothesis is in keeping with an extensive literature indicating that the functional lesions observed in the spermatozoa of infertile men, are commonly associated with signs of oxidative stress [1].

### ROS generation in spermatozoa

Oxidative stress, and its role in the origins of male infertility was first appreciated in 1943, when the Scottish andrologist John MacLeod demonstrated that catalase could support the motility of human spermatozoa incubated under aerobic conditions [15]. His explanation for these findings, that human spermatozoa are vulnerable to oxidative stress created by reactive oxygen species (ROS) such as H_2_O_2_, has been confirmed in a number of independent studies [1]. Human spermatozoa are capable of generating ROS and this activity is of physiological significance in promoting the tyrosine phosphorylation events associated with sperm capacitation. The ability of ROS to enhance the tyrosine phosphorylation status of human spermatozoa depends partly on the ability of H_2_O_2 _to suppress tyrosine phosphatase activity, and partly on the ability of these molecules to stimulate cAMP generation by the soluble form of adenylyl cyclase (sAC) [16,17]. The cAMP generated in this manner then stimulates tyrosine phosphorylation via a PKA dependent mechanism involving an, as yet, uncharacterised intermediary tyrosine kinase [17]. Redox control of tyrosine phosphorylation during sperm capacitation has been recorded for a large number of species including the rat [18], mouse [19], human [17], bull [16] and stallion [20].

This redox drive to capacitation involves a low, steady state level of ROS production. However, if, for any reason, this physiological rate of ROS generation should increase, or the spermatozoa should become exposed to exogenous ROS generated by, for example, infiltrating leukocytes, then a state of oxidative stress can be readily induced. Spermatozoa are particularly susceptible to such stress as a consequence of their high unsaturated fatty acid content and their limited store of antioxidant enzymes such as superoxide dismutase or glutathione peroxidase [1]. In keeping with this concept, exposure of human spermatozoa to ROS generated by xanthine oxidase disrupts the functional competence of spermatozoa at levels that have little impact on somatic cells and, importantly, this effect can be reversed by the addition of catalase [21,22]. Of interest in the context of this review, is that this same system has been used to demonstrate the damaging effect of ROS on nuclear DNA in spermatozoa [23]. Direct exposure of human spermatozoa to ROS disrupts not only the functional competence of these cells but also their genomic integrity [24]. In relation to the paternal origins of disease, it is significant that endogenous ROS generated by human spermatozoa can, and does, affect sperm function and DNA integrity [6]. The current debate centers on how such oxidative stress is created.

### Monitoring ROS production by spermatozoa

Following the initial report from Macleod [15], Aitken and Clarkson [25] went on to interpret the high levels of luminol-dependent chemiluminescence they observed in the spermatozoa of infertile patients, as evidence for ROS generation by such cells. The subpopulations of human spermatozoa responsible for this luminol-dependent activity have subsequently been isolated in the low density region of Percoll gradients [26,27] and linkages established with the aberrant retention of excess residual cytoplasm during spermiogenesis [27,28].

To further understand the biochemical basis of ROS generation in spermatozoa, attention soon focused on the use of other ROS-detecting probes apart from luminol. Owing to its sensitive nature, a popular reagent to use in this context was lucigenin. Lucigenin has been frequently deployed for the detection of ROS and is certainly capable of detecting superoxide anion (O_2_^-•^) [29,30]. For example, this probe has been used to detect NADPH oxidase type 2 (NOX2) -dependent O_2_^-• ^production in phagocytic cells and in cell-free ROS-generating systems, such as xanthine plus xanthine oxidase [31]. In the context of spermatozoa, lucigenin as been used successfully to detect the generation of O_2_^-• ^in rat sperm suspensions isolated from the cauda epididymides [32]. This signal was shown to be of mitochondrial origin, being inhibited by rotenone and stimulated by lactate, succinate and malate [32]. Although this is very good evidence for the production of mitochondrial ROS generation by rat spermatozoa, it is uncertain as to whether mitochondria are an important source of ROS in the spermatozoa of other species, particularly the human [25].

Notwithstanding the fact that mitochondrial production of ROS in spermatozoa remains largely unexplored, Vernet et al. [32] generated additional evidence for the non-mitochondrial production of ROS by rat spermatozoa. By isolating rat sperm membranes and adding lucigenin together with NADPH as a co-factor, they were able to show definitive chemiluminescent signals in this model system. This signal was inhibited with superoxide dismutase (SOD), DPI (diphenylene iodonium) and zinc [32]. Furthermore, addition of NADPH together with lucigenin to suspensions of human [33], mouse [34] rat [32] wallaby [35] and stallion [36] spermatozoa demonstrated that this lucigenin-dependent redox activity is a ubiquitous feature of mammalian spermatozoa.

**Figure 1:**
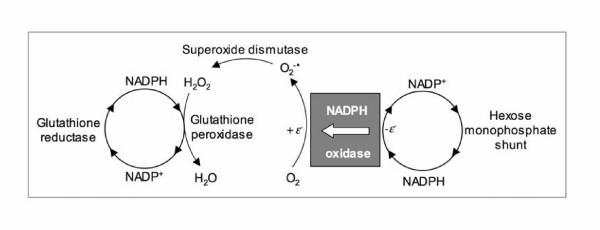
Schematic representation of NAD(P)H oxidase activity. This enzymes transfers electrons from NAD(P)H to ground state oxygen to create the superoxide anion radical. The latter then dismutates to hydrogen peroxide under the influence of superoxide dismutase. The hydrogen peroxide is predominantly scavenged by glutathione peroxidase, since human spermatozoa possess little catalase activity. Once this peroxidase activity is overwhelmed, a state of oxidative stress may be induced that disrupts the fertilizing capacity of the spermatozoa and the integrity of their DNA.

**Figure 2:**
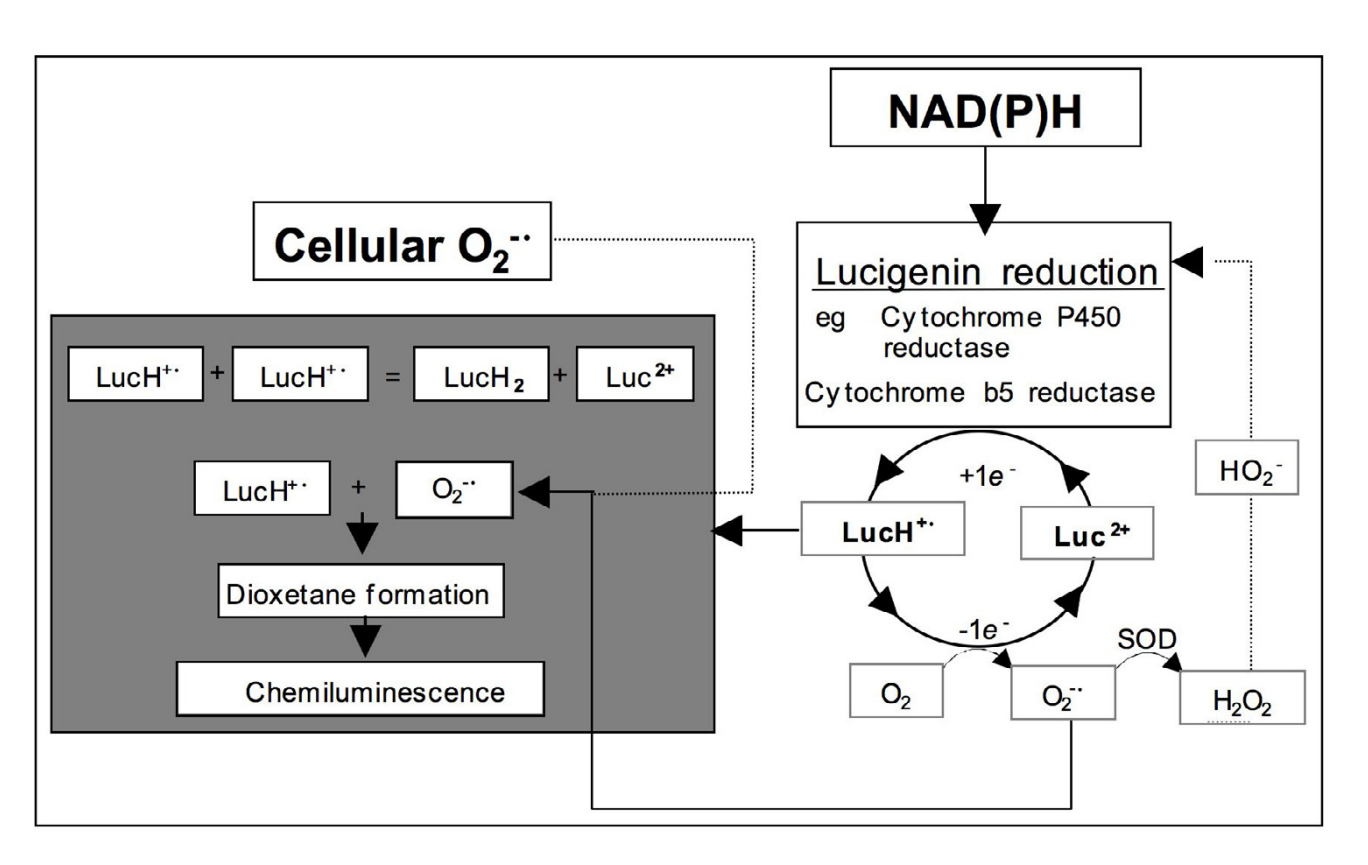
Schematic representation of the underlying chemistry of lucigenin chemiluminescence. Luc^2+ ^= lucigenin; LH^+• ^= a lucigenin radical created by the one electron reduction of Luc^2+^. The reaction of LH^+• ^with oxygen generates O_2_^-•^. The latter then participates in an oxygenation reaction with LH^+• ^generating a dioxetane that decomposes with the generation of chemiluminescence. Any entity that can bring about the one electron reduction of lucigenin can potentially create a redox cycle in the presence of oxygen that produces high levels of O_2_^-• ^and chemiluminescence. It is impossible to distinguish the relative contribution of such probe-dependent and cell-dependent chemiluminescence. Hence data obtained with this probe should be interpreted with caution.

In the case of human spermatozoa, NADPH-dependent lucigenin chemiluminescence was of non-mitochondrial in origin, being insensitive to rotenone, antimycin A, carbonyl cyanide m-chlorophenylhydrazone and sodium azide [33]. The initial interpretation of these data was that they represented support for the presence of an NADPH-oxidase in spermatozoa that could be responsible for the high rates of NAD(P)H-induced, lucigenin-dependent chemiluminescence recorded in defective sperm populations [37]. The general concept of such a theoretical oxidase is presented in Fig. 1. In essence, the enzyme serves to transfer electrons from NAD(P)H to ground state oxygen to create O_2_^-• ^that then dismutates to H_2_O_2 _under the influence of intracellular superoxide dismutase (Fig. 1). The interpretation of these NADPH-induced, lucigenin-dependent chemiluminescence signals is complex however [38], because of the tendency of this probe to redox cycle as a result of one-electron reductions conducted by enzymes, such as cytochrome-P450 reductase [39]. This scheme proposes the following steps: (i) a one electron reduction of lucigenin (L) under the influence of cytochrome P450-reductase and NADPH to generate a radical species (LH^+•^), (ii) a reaction between the latter and ground state oxygen to produce O_2_^-• ^and recycle the lucigenin back to its native state (L), (iii) and finally a reaction between LH^+• ^and O_2_^-•^, generated as a result of the redox cycling of lucigenin, to produce a dioxetane that, in turn, decomposes with the generation of chemiluminescence (Fig. 2).

As a consequence of this chemistry, O_2_^-•^-dependent, SOD-sensitive chemiluminescence can be generated in cellular systems that do not generate ROS themselves but are simply capable of redox-cycling the probe (lucigenin) used to detect this activity. In a similar fashion, NADH-induced lucigenin-dependent chemiluminescence has been shown to be associated with another intracellular reductase, cytochrome b5-reductase (CYB5R) in spermatozoa [40]. Thus, CYB5R was shown to co-elute from an anion exchange column with NADH-induced, lucigenin-dependent chemiluminescence activity, while over-expression of this same enzyme led to a 3-fold increase of this activity in COS7 cells. Although CYB5R is capable of reducing lucigenin in the presence of NADH, a paradox has arisen from these studies in that CYB5R should be inhibited by DPI. However, the NADH-dependent enzyme responsible for lucigenin reduction in spermatozoa is not inhibited with this reagent. Two explanations for this discrepancy are worthy of consideration. First, it is plausible that DPI penetration to the sites of lucigenin action may be limited in intact cells (this would explain why DPI was so much more effective in suppressing the lucigenin chemiluminescence observed in cell-free partially purified CYB5R preparations, compared with transiently transfected intact cells [35]) or alternatively, a second DPI-insensitive enzyme system may exist in whole cells, that is also capable of activating lucigenin.

Although this general redox cycling concept (Fig. 2) seems to explain how SOD-inhibited NAD(P)H-dependent chemiluminescence can be generated in the absence of primary O_2_^-• ^production, it has also been argued that the reaction between LH^+• ^and O_2 _is thermodynamically unlikely [41] and that redox cycling of this probe cannot occur in biological systems. In light of such reservations, we cannot be certain of the extent to which the elevated NAD(P)H-induced lucigenin signals detected in defective human spermatozoa [37] reflect primary O_2_^-• ^production.

### NAD(P)H-induced chemiluminescence and oxidative stress

**Figure 3:**
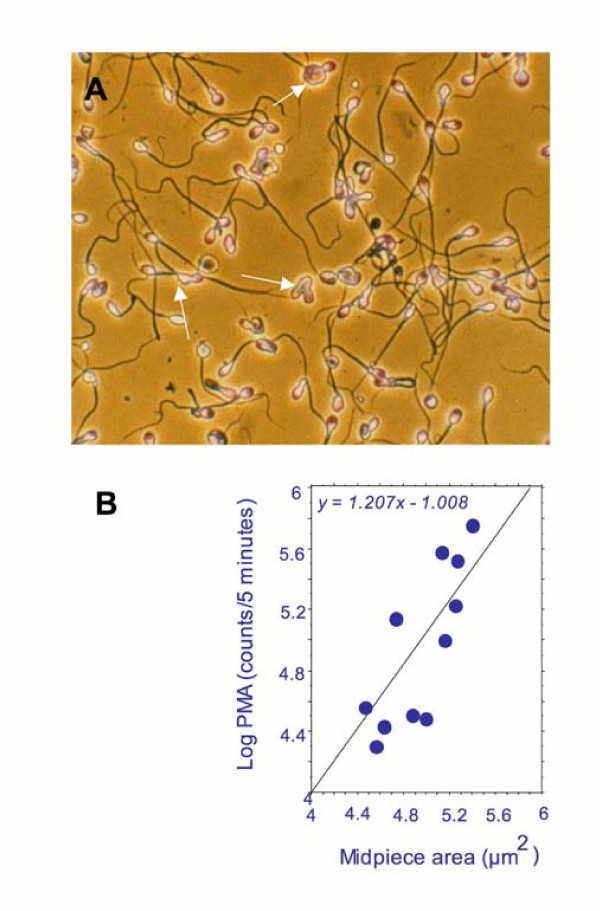
Errors during spermiogenesis can lead to the retention of excess residual cytoplasm by human spermatozoa. A) arrows point to cells possessing an irregular cytoplasmic mass in the neck region of the spermatozoon; significantly, human spermatozoa have lost the ability, possessed by most other mammalian species, to create a cytoplasmic droplet which is later discharged from the cell B) The amount of cytoplasm retained by human spermatozoa is highly correlated with the ability of leukocyte-free sperm suspensions to generate a chemiluminescence response to 12-myristate, 13-acetate phorbol ester (PMA), using luminol-peroxidase as the detection system.

Even if NAD(P)H-induced lucigenin-dependent chemiluminescence does simply reflect the presence of oxidoreductases capable of initiating the redox cycling of the probe, rather than primary O_2_^-• ^generation, the diagnostic significance of this activity may still reside in its ability to reflect oxidative stress in human sperm populations [37,42,43]. Thus, if the inter-individual differences in lucigenin chemiluminescence reflect the varying availability of certain reductases, this must, in turn, reflect inter-individual differences in the retention of residual cytoplasm during spermiogenesis. Such a conclusion would be in keeping with a large number of studies indicating that defective sperm function is positively correlated with the presence of numerous cytosolic enzymes that are markers of the cytoplasmic space, including lactic acid dehydrogenase, creatine kinase, SOD and glucose-6-phosphate dehydrogenase [6]. These observations, in turn, reflect the fact that defective sperm function is frequently associated with the retention of excess residual cytoplasm as a result of impaired cytoplasmic extrusion during spermiogenesis. Human spermatozoa are unusual in that they have lost the ability shown by most mammalian species to remodel any residual cytoplasm into a cytoplasmic droplet that is ultimately discharged from the cells either during epididymal maturation or at ejaculation. As a consequence, any residual cytoplasm that remains after spermiogenesis has been completed in the human, is retained by the spermatozoa as an amorphous cytoplasmic mass in the neck region of the cell (Fig. 3). Retention of this excess cytoplasm has been associated with the existence of oxidative stress in the germ line in several independent studies [27,28].

An explanation for this association between excess cytoplasmic retention and ROS production has been put forward, based on the correlation between the latter and the cellular content of glucose-6-phosphoate dehydrogenase in human sperm suspensions [28]. This enzyme regulates the rate of glucose flux through the hexose monophosphate shunt and is, in this way, responsible for controlling the intracellular availability of NADPH (Fig. 1). The latter may then serve as a substrate for putative ROS-generating enzymes in the germ line such as NOX 5 [44], and thereby, elicit a state of oxidative stress.

Although this explanation is consistent with the data generated in numerous independent studies, it still has the status of an unproven hypothesis rather than an experimentally proven fact. Definitive evidence for the presence of NOX 5 in human spermatozoa has still not been obtained and we still await independent confirmation of the ability of NOX 5 to generate ROS in spermatozoa. It has even been questioned whether human spermatozoa produce ROS from any source. Using the ability of dihydroethidium to react with O_2_^-• ^and produce 2OH ethidium we have recently secured incontrovertible evidence that human spermatozoa do in fact generate this free radical species and that the cellular production of O_2_^-• ^is significantly elevated in populations of defective sperm populations. Furthermore, the data we have generated to date suggests that this is not a consequence of defective mitochondrial function since a variety of mitochondrial inhibitors fail to disrupt the 2OH ethidium signal.

Such results suggest there must be a source of O_2_^-• ^in human spermatozoa that is extra-mitochondrial. A number of possibilities exist in this context. First of all, the above-mentioned NADPH-oxidase, NOX5, originally identified by Banfi et al. in human testes [44,45], has the potential to generate free radicals in a calcium-dependent manner, as originally described by Aitken and Clarkson [25]. Although the presence of NOX5 in human spermatozoa has not yet been confirmed, a recent publication in the mouse [46] employed Western blot analysis to record the presence of proteins exhibiting cross reactivity with antibodies against various components of the leukocyte NADPH oxidase complex (NOX2) including gp91phox, p67(phox), p47 phox and p40 (phox). The authors claim that this unusual oxidase is regulated by the availability of p40 (phox) and is independent of p22 (phox). They also assert that this oxidase is maximally active in testicular spermatozoa but decreases in concert with sperm maturation. Confirmation of this pattern of NADPH oxidase activity is strategically important since it would add weight to the argument that ROS production by human spermatozoa is an inverse function of their state of functional maturity.

In addition to NADPH oxidase activity, it is also clear that spermatozoa will generate ROS when placed in contact with certain xenobiotics. Examples of such compounds include endocrine disruptors with estrogenic properties that are capable of inducing ROS production by male germ cells and initiating free radical-mediated DNA damage [47]. Similarly, oxidative stress and DNA damage can be induced in spermatozoa via metal-catalysed redox activity involving, for example nickel [48] or iron [49] as well as phthalate esters [50].

Another potential source of ROS in human spermatozoa is a trans-plasma membrane oxidoreductase system that removes electrons from NAD(P)H on the cytoplasmic surface of the cell and transfers them to oxygen on the outer leaf of the plasma membrane via intermediate carriers such as ubiquinone. Evidence to support the existence of such systems in somatic cells, arises from the ability of the latter to reduce artificial membrane-impermeant electron acceptors such as potassium ferricyanide, in concert with the concomitant oxidation of cytosolic NADH [51]. Interestingly, the activity of such plasma membrane oxidase systems has been demonstrated to increase in Rho O cells that lack functional mitochondria [51,52]. In such cases, up-regulation of the plasma membrane redox system may help maintain an adequate pool of NAD+ to fuel the increased glycolysis needed to maintain cell viability in the absence of mitochondrial activity. Human spermatozoa have been shown to possess redox activity typical of such plasma membrane electron transport chains [32,53]. Thus, human spermatozoa possess a capacity to reduce the probe WST-1 in the presence of an intermediate electron acceptor in a similar fashion to the plasma membrane redox system described in somatic cells by Berridge and Tan [54,55]. This transmembrane electron transfer system in spermatozoa shares similarities with the Berridge and Tan activity in being inhibited by SOD, capsaicin, (a potent vanilloid inhibitor) and N-ethyl maleimide (NEM, a membrane permeant alkylating agent). However, the susceptibility of the sperm oxidase activity to the membrane impermeant thiol blocking agent pCMBS (p-chloromercuriphenylsulphonate) as well as retinoic acid, distinguishes the sperm-based activity from that detected in somatic cells [37]. Since mitochondrial function is frequently defective in populations of human spermatozoa [56], it is possible that this trans-plasma membrane redox system is up-regulated in defective human spermatozoa in a similar fashion to the enhanced activity recorded in Rho O cells in order to maintain the redox status of the NAD^+^/NADH couple. Further characterization of this putative electron transport chain is clearly warranted. However, this task will not be easy given that, by definition, the redox activity ascribed to such systems depends on the close interaction of several independent constituents, not on a single identifiable entity.

Another form of oxidase activity detected by Berridge and Tan [54,55] is a superficial enzyme that removes electrons from exogenously applied sources of reducing equivalents (eg. NAD(P)H) to generate O_2_^-•^. Exogenous NAD(P)H will certainly reduce extracellular electron acceptors such as WST-1 in the presence of human spermatozoa [37]. Moreover, the susceptibility of such activity to inhibition with pCMBS corresponds to the activity detected in a variety of cell types, including HeLa and Jurkat cells, by Berridge and Tan [54,55]. However, the activity elicited in the presence of spermatozoa could be distinguished from that generated by somatic cells by virtue of the lack of stimulation observed with NEM [37,54,55].

Nitric oxide (NO) is another oxygen free radical which is apparently generated by defective populations of human spermatozoa. NO is normally generated from L-arginine by three isoforms of nitric oxide synthase (NOS). Recent mouse knock out experiments indicate that the selective deletion of these NOS isoforms has no impact on the ability of the spermatozoa to achieve fertilization. In other words, NO does not appear to have a positive role to play in the generation of functional gametes [57]. However the fact that iNOS deficient spermatozoa exhibited significantly higher in vitro fertilization rates than the wild-type controls, clearly suggests that NO may be involved in the etiology of defective sperm function. NO clearly has a detrimental effect on normal sperm function inhibiting both motility and the competence of these cells for sperm-zona binding [58]. Moreover the NO levels in seminal plasma are negatively correlated with sperm movement in human semen samples [59]. The source of this NO is still an open question. The involvement of NOS is suggested by the negative correlation observed between the pattern of NOS expression on human spermatozoa and percentage motility [60]. However, it has also been pointed out the NO and peroxynitrite formation can be stimulated in mammalian spermatozoa using D-arginine, which cannot be a substrate for NOS. Under these circumstances it is possible that NO is being generated non-enzymatically through an H_2_O_2_-mediated attack on arginine [61].

Clearly there are many potential sources of ROS in the male germ line. The task that now confronts us is to determine which of these multifarious sources are responsible for the oxidative stress observed in the spermatozoa of male patients.

## Conclusion

Human spermatozoa are redox active cells that are capable of generating O_2_^-• ^and H_2_O_2_. This activity is of fundamental biological importance in regulating the signal transduction pathways that control sperm capacitation. However, excess exposure to ROS can lead to pathological damage to human spermatozoa curtailing their competence for fertilization and disrupting their genetic integrity. DNA damage in these cells appears to be largely oxidative and is associated with a wide variety of adverse outcomes including impaired conception rates, increased incidences of abortion and defects in the offspring, including childhood cancer and dominant genetic diseases such as achondroplasia. It is hypothesized that such effects in the F1 generation involve the aberrant repair of oxidative DNA damage in the newly fertilized zygote. The etiology of oxidative stress in the male germ line is being actively researched at the present time. While spermatozoa certainly generate ROS, the biochemical basis of this activity is uncertain and may be multifactorial. Errors of spermiogenesis associated with the retention of excess residual cytoplasm appear to be associated with oxidative stress as a consequence of enhanced ROS production by uncharacterised 'oxidases', plasma membrane electron transport chains or oxidoreductases capable of activating redox-cycling xenobiotics. Electron leakage from defective sperm mitochondria represent yet another potential source of oxygen radicals. Given the clinical significance of oxidative stress in human spermatozoa, resolving the biochemical basis of this condition is a high priority task for the future.
